# Identification of brain-enriched proteins in CSF as biomarkers of relapsing remitting multiple sclerosis

**DOI:** 10.1186/s12014-024-09494-5

**Published:** 2024-06-16

**Authors:** Lincoln I. Wurtz, Evdokiya Knyazhanskaya, Dorsa Sohaei, Ioannis Prassas, Sean Pittock, Maria Alice V. Willrich, Ruba Saadeh, Ruchi Gupta, Hunter J. Atkinson, Diane Grill, Martin Stengelin, Simon Thebault, Mark S. Freedman, Eleftherios P. Diamandis, Isobel A. Scarisbrick

**Affiliations:** 1 Medical Scientist Training Program, Mayo Clinic, Rochester, MN USA; 2 Mayo Clinic Alix School of Medicine, Mayo Clinic, Rochester, MN USA; 3 Department of Laboratory Medicine and Pathobiology, University of Toronto, Toronto, Canada; 4 Mount Sinai Hospital, Toronto, Canada; 5 Laboratory Medicine Program, University Health Network, Toronto, Canada; 6 Department of Neurology, Mayo Clinic, Rochester, MN USA; 7 Center for Multiple Sclerosis and Autoimmune Neurology, Mayo Clinic, Rochester, MN USA; 8 Department of Laboratory Medicine and Pathology, Mayo Clinic, Rochester, MN USA; 9 Department of Quantitative Health Sciences, Mayo Clinic, Rochester, MN USA; 10 Meso Scale Diagnostics, LLC. (MSD), Rockville, MD USA; 11 Department of Medicine and The Ottawa Research Institute, Ottawa, Canada; 12 Division of Multiple Sclerosis, Department of Neurology, The University of Pennsylvania, Philadelphia, USA; 13 Lunenfeld-Tanenbaum Research Institute (LTRI), Sinai Health System, Toronto, Canada; 14 Department of Physical Medicine and Rehabilitation, Mayo Clinic, Rochester, MN 55905 USA

**Keywords:** Multiple sclerosis, CSF, Biomarkers, Proteomics

## Abstract

**Background:**

Multiple sclerosis (MS) is a clinically and biologically heterogenous disease with currently unpredictable progression and relapse. After the development and success of neurofilament as a cerebrospinal fluid (CSF) biomarker, there is reinvigorated interest in identifying other markers of or contributors to disease. The objective of this study is to probe the predictive potential of a panel of brain-enriched proteins on MS disease progression and subtype.

**Methods:**

This study includes 40 individuals with MS and 14 headache controls. The MS cohort consists of 20 relapsing remitting (RR) and 20 primary progressive (PP) patients. The CSF of all individuals was analyzed for 63 brain enriched proteins using a method of liquid-chromatography tandem mass spectrometry. Wilcoxon rank sum test, Kruskal-Wallis one-way ANOVA, logistic regression, and Pearson correlation were used to refine the list of candidates by comparing relative protein concentrations as well as relation to known imaging and molecular biomarkers.

**Results:**

We report 30 proteins with some relevance to disease, clinical subtype, or severity. Strikingly, we observed widespread protein depletion in the disease CSF as compared to control. We identified numerous markers of relapsing disease, including KLK6 (kallikrein 6, OR = 0.367, *p* < 0.05), which may be driven by active disease as defined by MRI enhancing lesions. Other oligodendrocyte-enriched proteins also appeared at reduced levels in relapsing disease, namely CNDP1 (carnosine dipeptidase 1), LINGO1 (leucine rich repeat and Immunoglobin-like domain-containing protein 1), MAG (myelin associated glycoprotein), and MOG (myelin oligodendrocyte glycoprotein). Finally, we identified three proteins—CNDP1, APLP1 (amyloid beta precursor like protein 1), and OLFM1 (olfactomedin 1)—that were statistically different in relapsing vs. progressive disease raising the potential for use as an early biomarker to discriminate clinical subtype.

**Conclusions:**

We illustrate the utility of targeted mass spectrometry in generating potential targets for future biomarker studies and highlight reductions in brain-enriched proteins as markers of the relapsing remitting disease stage.

**Supplementary Information:**

The online version contains supplementary material available at 10.1186/s12014-024-09494-5.

## Background

Multiple sclerosis (MS) is an immune-mediated disease of the central nervous system (CNS) characterized by demyelination of axons resulting in white matter lesions and, eventually, neuronal degeneration. Broadly, MS patients can be subtyped into relapsing remitting (RR) disease, where bouts of inflammation associate with clinical attacks, vs. primary progressive (PP), where overt clinical attacks are less prominent and neurological disability accumulates insidiously. However, these distinctions are mostly clinical with limited evidence indicating distinctive disease processes, and it is increasingly recognized that both relapsing and progressive biologies are present to different degrees from disease onset in all individuals [1, 2]. Further, both the pathological lesions and clinical presentation in cases of MS are highly variable [3]. This poses a clinical management challenge at the time of diagnosis as it is difficult to predict imminent relapse, response to therapy, and disability worsening. A non-specific biomarker of neuroaxonal damage, neurofilament light chain (NF-L), has generated excitement as it has the potential to address some of these challenges [4–7]. Just as NF-L has proven useful for MS management, other brain-enriched proteins may share some or all these biomarker qualities, yet they remain largely unexplored.

Cerebrospinal fluid (CSF) is particularly useful for exploring proteins of neural or glial origin due to its intimate proximity and the ability to reflect protein concentrations of the brain parenchyma [8, 9]. While collection of CSF is more invasive and cumbersome than blood draws, it remains the safest portal into the brain proteome. Further, clinical evaluation of patients with suspected MS may already include CSF sampling for oligoclonal bands, so additional analysis of CSF targets fits easily into clinical workflow.

Bottom-up mass spectrometry-based proteomics is useful in detecting and quantifying multiple CSF proteins at once. Targeted approaches using a panel of proteins and their known fragmentation pattern can be used to achieve high throughput in a single sample in a process called parallel reaction monitoring (PRM). Diamandis et al. developed a liquid chromatography-tandem mass spectrometry assay (LC/MS/MS) to monitor a panel of brain-related proteins in the CSF using a relatively small CSF volume [10–13].

Here, we applied a targeted proteomic method to study the putative value of a panel of 63 brain-associated proteins as biomarkers in multiple sclerosis, and two broad clinical subtypes of relapsing remitting (RR) and primary progressive (PP) disease. We evaluated each protein for its ability to predict disease, discriminate clinical subtypes, and its correlation to commonly used biochemical, imaging, and clinical markers of disease. In this preliminary study, we found 30 distinct proteins with disease relevance. This includes 13 proteins with reduced expression in MS, 20 in RR, one in PP, and one, CNDP1, showed lower expression in RR as compared to PP subtypes. Many of these markers also showed the potential to discriminate disease or subtype after controlling for age. Additionally, oligodendrocyte-enriched proteins like KLK6, MAG, and MOG were differentially expressed in active disease as compared to control CSF. In sum, our work provides crucial proof of concept for the PRM method applied to neurological disease and identifies a list of proteins deserving of future study.

## Methods

### Study population and sample collection

A cohort of cerebrospinal fluid residual samples from consecutive patients with clinically ordered laboratory tests for oligoclonal banding and CSF IgG index were banked for biomarker discovery and chart-reviewed by a neurologist, after Mayo Clinic Institutional Review Board approval (IRB #15–000480). Chart review was conducted manually by reviewing each patient’s electronic health record, and included demographic, clinical, radiology and laboratory testing information from all patients available at the time of the sample collection. Diagnosis of MS was based in the 2010 McDonald criteria, hence oligoclonal banding test results were not used as part of the definition of MS. MS included patients with RR, PP, SP, tumefactive MS, clinically isolated syndrome (CIS) and radiologically isolated syndrome (RIS). CSF samples from patients with a clinical diagnosis of RR (*n* = 20) or PP (*n* = 20) were hand selected. In addition, patients with a chief complaint of headache and no other reason for oligoclonal banding test in their charts were used as controls (*n* = 14). No follow-up information was collected. Samples were collected as part of routine clinical procedure and transported to clinical laboratories via pneumatic tubes, often reaching central processing area in less than one hour post draw. If samples were not immediately sent to the clinical laboratory, they were kept frozen at -20 °C overnight. Samples were kept frozen at -80 °C and had undergone two freeze-thaw cycles by the time of this study.

### Sample processing

Patient CSF samples were coded, devoid of diagnosis and sent to Mount Sinai Hospital in Toronto for protein purification and analysis on LC-MS/MS with EASY-nLC 1000 and Q-Exactive HF-X with nanospray ionization. XCalibur software was used to determine peak integration. Relative protein quantification is reported as AUClight/AUCheavy, where AUC stands for area under the curve. Clinical samples were analyzed in duplicate and diagnosis remained unknown to experimenters prior to statistical analysis.

### Parallel reaction monitoring assay

A parallel reaction monitoring (PRM) assay was developed to simultaneously quantify 63 brain related proteins as described previously, most recently in Sohaei (2023) [13]. This manuscript includes 66 peptides representing 63 distinct proteins. Peptides were excluded from analysis if they had zero expression in at least a third of samples or showed non-variability, leaving 54 for further analysis.

### Mass spectrometry sample preparation

The protocol for CSF sample preparation used in this study by our collaborators was previously published [10–13]. In brief, 15 µg of total CSF protein per patient was used for protein digestion. Samples were first denatured using 0.05% RapiGest (Waters, USA) and reduced with 5 mM dithiothreitol (DTT) (Sigma-Aldrich, Canada) at 60 °C for 40 min. Then, the samples were alkylated with 15 mM iodoacetamide (IAA) (Sigma-Aldrich, Canada) for 60 min at room temperature before trypsin digestion (at a ratio of 1:30 trypsin to total protein). The following day, trifluoroacetic acid (Sigma-Aldrich, Canada) was added to a final concentration of 1% and placed on a shaker at 37 °C for 40 min. After centrifugation at 13,000 g for 30 min, the supernatant was retained. At this point, a mixture of 68 isotopically labeled peptides (52 candidates from a previous study [11, 12], and C1QTNF4, CDH18, CDH8, GPR158, IGLON5, LGI1, MDGA2, PCDH8, PCDH9, PTPRN, TMEM132A, TMEM59L) was spiked into the digest. Peptides were then purified by extraction using OMIX C18 tips (Agilent Technologies, USA), eluted in 3 µL of buffer C (comprising 64.9% acetonitrile, 35% water, and 0.1% formic acid), and finally diluted with 57 µL of buffer A (0.1% formic acid).

### Liquid-chromatography-tandem mass spectrometry (LC-MS/MS)

Following sample preparation all samples were subjected to LC-MS/MS analysis, following our previously published targeted proteomic PRM pipelines [13]. In brief, 18 µL of sample was loaded onto a trap column (0.75 µm ID × 3.3 cm) using the EASY-nLC 1000 system (Thermo Fisher Scientific) with buffer A (0.1% formic acid). Peptides were then eluted onto an analytical column (0.75 µm ID × 15 cm) using an increasing concentration of buffer B (99.9% acetonitrile and 0.1% formic acid). Our liquid chromatography system was coupled online to a Q-Exactive HF-X mass spectrometer (Thermo Fisher Scientific) equipped with a nano-electrospray ionization source. A 60-minute PRM method was employed, with full MS1 scans acquired at a resolution of 120,000 and PRM MS/MS spectra at a resolution of 15,000. Automatic gain control settings were adjusted accordingly for MS1 and MS2. Data acquisition was performed in ‘profile’ mode with specific parameters for inclusion mass accuracy and scheduled duration for each peptide. Blinded clinical samples were analyzed in duplicate.

### Data analysis

The generation of raw files was conducted using XCalibur software version 4.3.73.11 on the Q-Exactive HF-X. Subsequently, these raw files were transferred to Skyline software version 20.1.0.155 for peak integration and quantification based on the area under the curve (AUC), as previously described [13].

#### Quality control

As quality control we prepared a CSF pool comprising 16 individual samples which we digested alongside clinical samples, with isotopically labeled peptides added. These control samples were analyzed before, during, and after the run to assess assay reproducibility by evaluating the L/H ratio for each peptide. Additionally, the stability of the LC-MS system was confirmed by periodically running bovine serum albumin, as previously described [13].

### Statistical analysis

The non-parametric Wilcoxon rank sum test was used for direct comparisons between two groups (e.g. MS vs. Ctrl, RR vs. Ctrl, PP vs. Ctrl, RR vs. PP), and the Kruskal-Wallis one-way nonparametric analysis of variance was used to test for differences between more than two groups (e.g. Ctrl vs. RR vs. PP).

Logistic regression was used to evaluate the age-adjusted effect of each protein on the comparison groups (e.g. MS vs. Ctrl). Specifically, the comparison group was the response variable, and age was included in each model. Proteins were scaled by subtracting the mean and dividing by the standard deviation. Results from the logistic regression are presented in forest plots displaying the odds ratio for a one standard deviation change in each protein. The C-statistic from these models is reported as the AUC.

The relationship between protein concentration and expanded disability status scale (EDSS) was evaluated via linear regression. R^2^ values, which is the percent of variability explained by the regression, are presented as a measure of association. Spearman’s correlation was used for comparison between the proteins.

Analyses were conducted using the R statistical package v4.2.2 [14] and GraphPad Prism v. 9.5.1 for Windows (GraphPad Software, San Diego, CA, USA). Summary figures created in Biorender.

### Neurofilament light quantification

In addition to quantification by PRM assay, levels of neurofilament light (NF-L) in the CSF were separately quantified using R-PLEX Human Neurofilament L assay, Meso Scale Discovery (MSD); catalog # K1517XR [15]. CSF samples were measured at ten-fold dilution.

### Subcellular localization

Subcellular analysis localization based on COMPARTMENTS dataset [16] with a confidence of 4 or 5. Manual annotation was used if data was weak or not listed.

### STRING pathway analysis

Connected graph of the protein candidates was created using STRING v11 [17] using a full STRING network. Edges indicate confidence. Active interaction sources include text mining, experiments, databases, co-expression, neighborhood, gene fusion, and co-occurrence. Minimum required interaction score was set at medium confidence (0.400). K-means clustering was used with three clusters. Rstudio version 1.4.1103 was used with ggplot2 package to create String bar graphs in Figure 5.

## Results

### Baseline clinical and Demographic Data

**Fig. 1:**
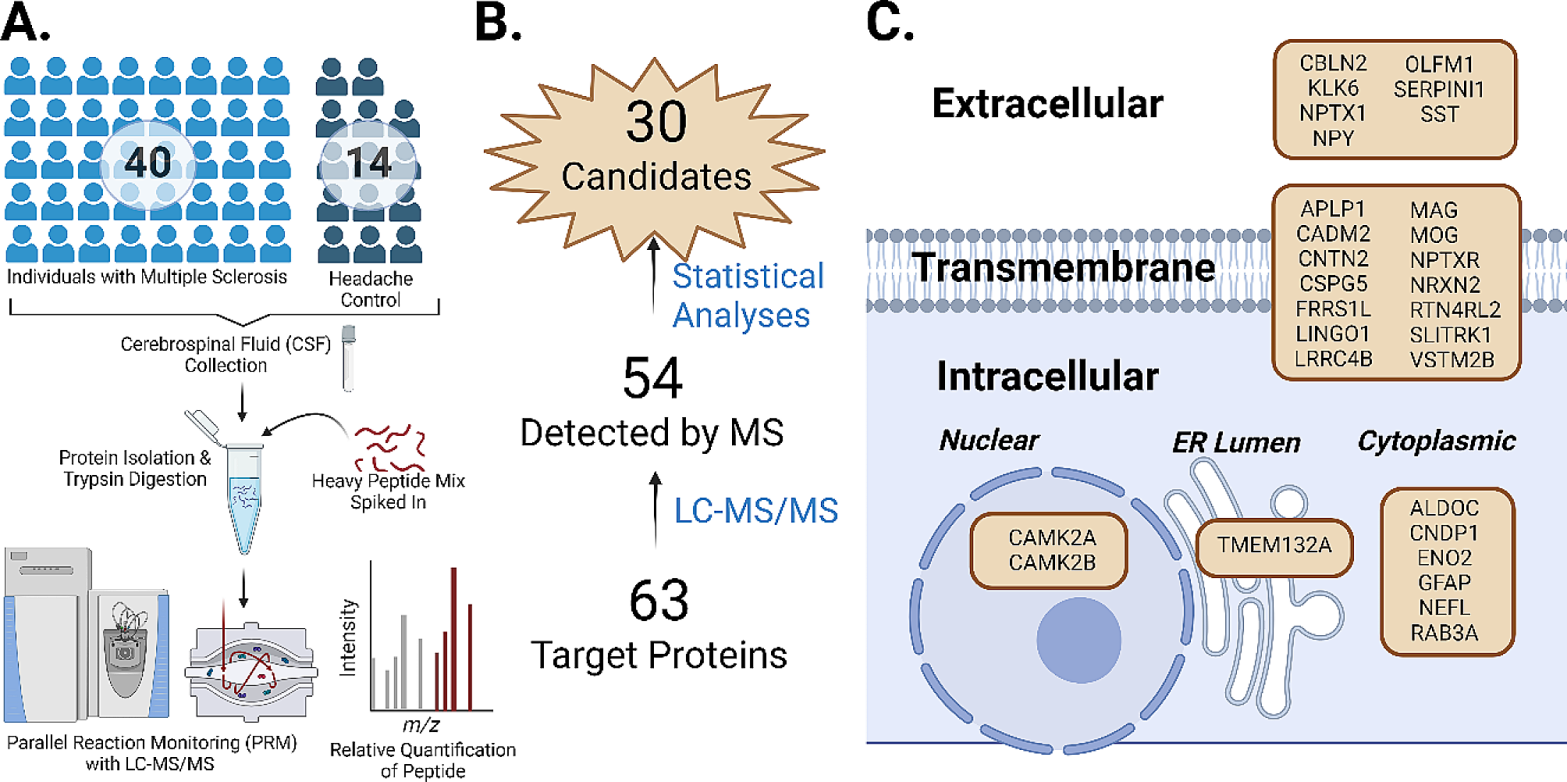
Study workflow, protein candidate identification, and subcellular localization. **(A)** Patient samples were collected via lumbar puncture, frozen, and sent to Mount Sinai Hospital for protein purification and analysis on LC-MS/MS. **(B)** Of the 63 target proteins, 54 were consistently detected by the LC-MS/MS and appropriate for analysis. Statistical testing including Wilcoxon p, Kruskal-Wallis, linear regression, logistic regression, or Mann-Whitney test refined the 54 target protein panel to a subset of 30 protein candidates. **(C)** Subcellular localization of the 30 protein candidates using COMPARTMENTS database indicates a predominance of membrane associated proteins.

The cerebrospinal fluid (CSF) of 40 individuals with clinically diagnosed multiple sclerosis (MS) at time of draw was compared to a control population of 14 patients undergoing diagnostic workup for headache (Fig. 1). The MS group can be further subcharacterized into 20 patients with either relapsing remitting multiple sclerosis (RR) or primary progressive (PP). There was a strong female bias in the RR-MS cases (75%), which is matched well with the control group (78.6%) (Table 1). In comparison, there were roughly equal numbers of males and females in PP-MS cases (55% female). These data reflect the diminished sex disparity in PP-MS observed in prior demographic studies [18]. The ages are also well matched between MS and control populations with a median age of 49.0 and 46.0, respectively.

The median time from symptom onset to CSF sample collection was 1.7 years in the relapsing remitting population and 4.0 years in the primary progressive group. Of those with MS, 34 (85%) had an expanded disability status scale (EDSS) score recorded at the time of CSF sampling. The median EDSS was 1.0 in the RR-MS population indicating no disability with minimal signs of disease [19]. For PP-MS, the median EDSS was 3.0, with males displaying a slightly higher disability (3.5) (see Additional File 1). Upon clinical review, we determined if each patient had at least one MRI enhancing lesion at the time of CSF draw (TOD). Out of the 40 patients with MS, 9 were classified as positive for enhancing lesions.

**Table 1:** Patient demographics and descriptive statistics. The CSF of 54 individuals was collected via lumbar puncture for downstream proteomic analysis. Of the 54 individuals in the study, 40 individuals had clinically diagnosed MS (65% female, median age of 49) of both subtypes (*n* = 20 RR, *n* = 20 PP). As a non-inflammatory neurological disorder control, 14 individuals with headache (78.6% female, median age of 46) were used. Expanded disability scale score (EDSS), time to sample collection after symptom onset, and whether a patient had enhancing lesions on MRI are also reported.

	Controls (*N* = 14)	RRMS (*N* = 20)	PPMS (*N* = 20)	All Cases (*N* = 40)
**Sex**				
Male	3 (21.4%)	5 (25.0%)	9 (45.0%)	14 (35.0%)
Female	11 (78.6%)	15 (75.0%)	11 (55.0%)	26 (65.0%)
**Age at Collection**				
Median	46.0	37.5	56.5	49.0
Q1, Q3	40.2, 52.0	17.0, 45.2	53.0, 62.2	37.8, 57.5
**EDSS at Sample Collection**				
N	0	19	15	34
Median	NA	1.0	3.0	1.5
Q1, Q3	NA	1.0, 1.5	2.0, 5.8	1.0, 3.0
**Time to Sample from Onset of Symptoms (yrs)**				
N	0	20	20	40
Median	NA	1.7	4.0	2.8
Q1, Q3	NA	0.6, 3.4	2.6, 6.7	1.1, 5.4
**MRI Enhancing Lesions at Sample Collection**				
N	10	20	20	40
No	10 (100.0%)	12 (60.0%)	19 (95.0%)	31 (77.5%)
Yes	0 (0.0%)	8 (40.0%)	1 (5.0%)	9 (22.5%)

### The CSF of individuals with MS is depleted in brain-enriched proteins

**Fig. 2:**
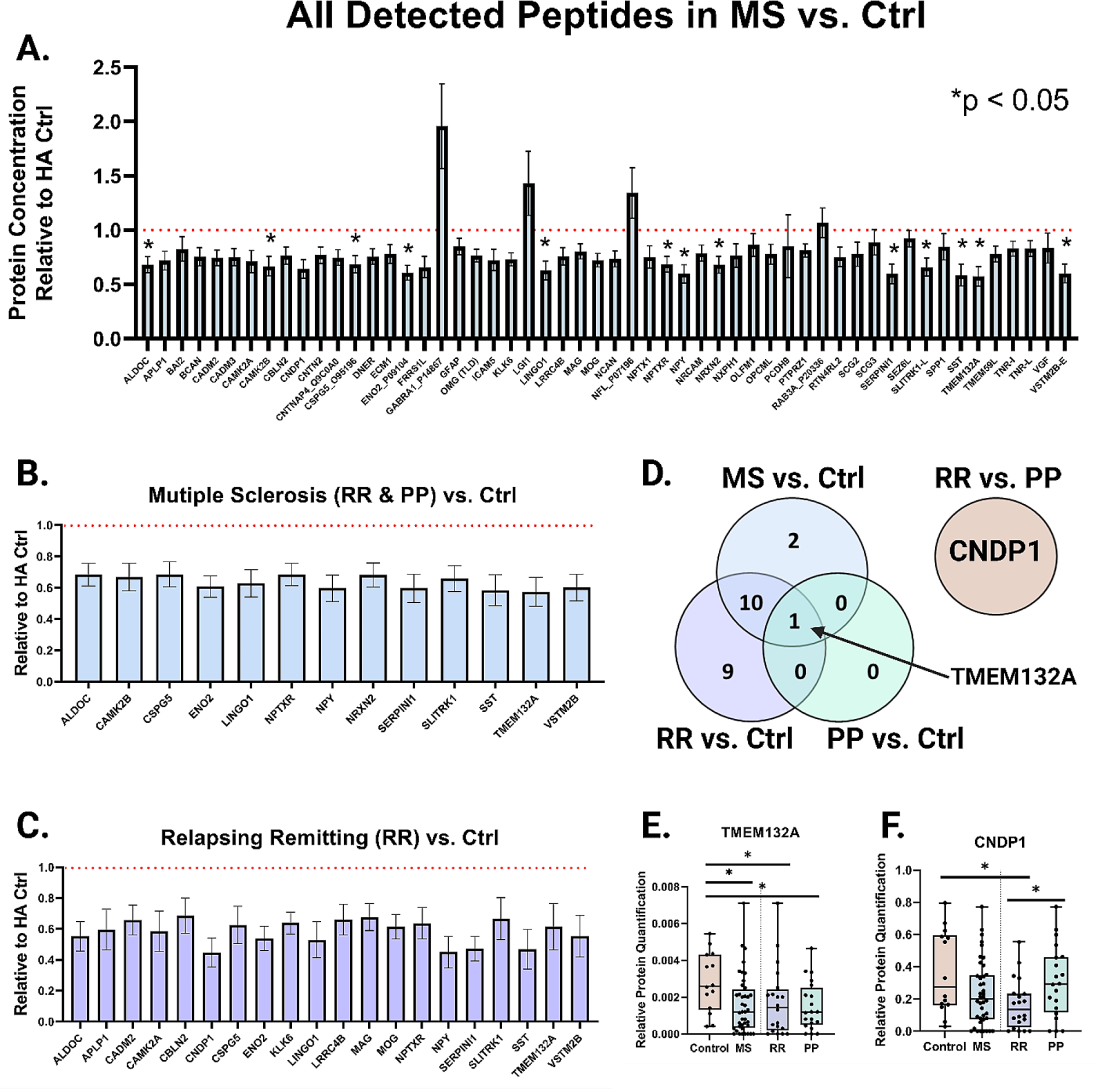
All significantly detected proteins have decreased expression in MS. **(A)** All 54 detected peptides by LC-MS/MS are displayed relative to control. Focused subset of proteins that are significant in either all MS patients combined vs. control **(B)** or relapsing remitting group vs. control **(C)**. **(D)** Venn-Diagram illustrating the statistically significant proteins for the following comparisons: MS vs. Ctrl, RR vs. Ctrl, and PP vs. Ctrl. TMEM132A was the only significant protein across all three analyses. CNDP1 was the only significantly different protein in RR vs. PP analysis. Box and whisker plots of the relative protein quantification of TMEM132A **(E)** or CNDP1 **(F)** in control, MS, RR, or PP groups. Box represents median, quartile 1, and quartile 3 with whiskers to range. Bar graphs represent mean with error bars +/- SEM. *p < 0.05 by Wilcoxon for all graphs in figure.

To determine how MS alters the CSF proteome, we measured a panel of 63-brain enriched proteins using parallel reaction monitoring on liquid chromatography tandem mass spectrometry (LC-MS) [10–13]. Of the 63 proteins studied, 54 were consistently detected via LC-MS and appropriate for analysis. First, we investigated differences in CSF protein targets between all individuals with MS regardless of subtype and headache control (MS vs. CTRL) (Table 2; Fig. 2A). All 13 significant differentially expressed proteins were decreased in the MS group by Wilcoxon test (Fig. 2B). If we directly compare RR vs. CTRL, we find there are 20 proteins differentially regulated in disease. Again, all 20 significant proteins are decreased in the RR group (Fig. 2C). Fewer proteins are differentially expressed between PP vs. Ctrl. Only a single protein achieves significance, TMEM132A (Table 2; Fig. 2E).

Next, we examined the difference between RR and PP and discovered CNDP1 was significantly lower in RR compared to PP. Of the panel of proteins analyzed, CNDP1 was the only protein that was significantly different between the two MS subtypes, prior to controlling for age (see below). In summary, the head-to-head statistical analysis outlined a subset of 22 proteins out of the original 63 analyzed that showed some level of discriminated expression in a disease state.

**Table 2:** Significantly different CSF proteins in MS, RR, or PP. Reported mean quantity (+/- standard deviation) of all proteins that showed significance by either Wilcoxon (*p < 0.05) or Kruskal-Wallis (**p < 0.05) statistical analysis. *p*-values < 0.05 are bolded.

	Mean Relative Protein Concentration (+/- SD)			*P*-value				
Gene Name	Ctrl (*n =* 14)	RR (*n* = 20)	PPMS (*n* = 20)	MS vs. CTRL*	RR vs. Ctrl*	PP vs. Ctrl*	RR vs. PP*	RR v. PP v. CTRL**
ALDOC	0.99 ± 0.33	0.55 ± 0.43	0.81 ± 0.47	**0.02**	**0.01**	---	---	**0.01**
APLP1	0.32 ± 0.14	0.19 ± 0.19	0.27 ± 0.16	---	**0.04**	---	---	---
CADM2	0.10 ± 0.05	0.07 ± 0.04	0.08 ± 0.05	---	**0.03**	---	---	---
CAM2KA	0.29 ± 0.15	0.17 ± 0.17	0.24 ± 0.20	0.05	**0.02**	---	---	0.05
CAM2KB	0.48 ± 0.24	0.30 ± 0.30	0.34 ± 0.25	**0.04**	---	---	---	---
CBLN2	0.02 ± 0.01	0.01 ± 0.01	0.02 ± 0.01	---	**0.04**	---	---	---
CNDP1	0.37 ± 0.26	0.16 ± 0.16	0.31 ± 0.22	---	**0.02**	---	**0.03**	**0.03**
CNTN2	0.33 ± 0.12	0.23 ± 0.14	0.27 ± 0.18	---	0.05	---	---	---
CNTNAP4	0.82 ± 0.37	0.55 ± 0.35	0.68 ± 0.40	---	0.05	---	---	---
CSPG5	1.95 ± 0.77	1.22 ± 1.06	1.45 ± 0.93	**0.04**	**0.04**	---	---	---
ENO2	0.05 ± 0.03	0.03 ± 0.02	0.03 ± 0.03	**0.04**	**0.03**	---	---	---
KLK6	0.53 ± 0.23	0.34 ± 0.17	0.44 ± 0.23	0.05	**0.02**	---	---	0.05
LINGO1	0.50 ± 0.25	0.27 ± 0.26	0.37 ± 0.29	**0.01**	**0.01**	0.05	---	**0.02**
LRRC4B	0.15 ± 0.06	0.10 ± 0.07	0.13 ± 0.08	---	**0.03**	---	---	---
MAG	0.38 ± 0.16	0.26 ± 0.15	0.35 ± 0.18	---	**0.04**	---	---	---
MOG	0.11 ± 0.05	0.07 ± 0.04	0.09 ± 0.04	0.05	**0.01**	---	---	**0.03**
NPTXR	0.23 ± 0.10	0.15 ± 0.11	0.17 ± 0.11	**0.02**	**0.02**	---	---	---
NPY	0.10 ± 0.06	0.04 ± 0.04	0.07 ± 0.06	**0.03**	**0.01**	---	---	**0.04**
NRXN2	2.12 ± 1.01	1.46 ± 1.15	1.44 ± 0.95	**0.03**	---	---	---	---
SERPINI1	0.09 ± 0.07	0.04 ± 0.03	0.06 ± 0.06	**0.03**	**0.01**	---	---	---
SLITRK1	0.05 ± 0.02	0.03 ± 0.03	0.03 ± 0.02	**0.03**	**0.04**	0.05	---	---
SST	0.06 ± 0.03	0.03 ± 0.03	0.04 ± 0.04	**0.02**	**0.01**	---	---	**0.03**
TMEM132A	0.003 ± 0.002	0.002 ± 0.002	0.002 ± 0.001	**0.01**	**0.03**	**0.02**	---	**0.04**
VSTM2B	0.001 ± 0.001	0.001 ± 0.001	0.001 ± 0.001	**0.02**	**0.03**	---	---	0.05

* Wilcoxon *p* value

** Kruskal-Wallis *p* value

### Select proteins hold potential to predict disease or disease state even after controlling for age

**Fig. 3:**
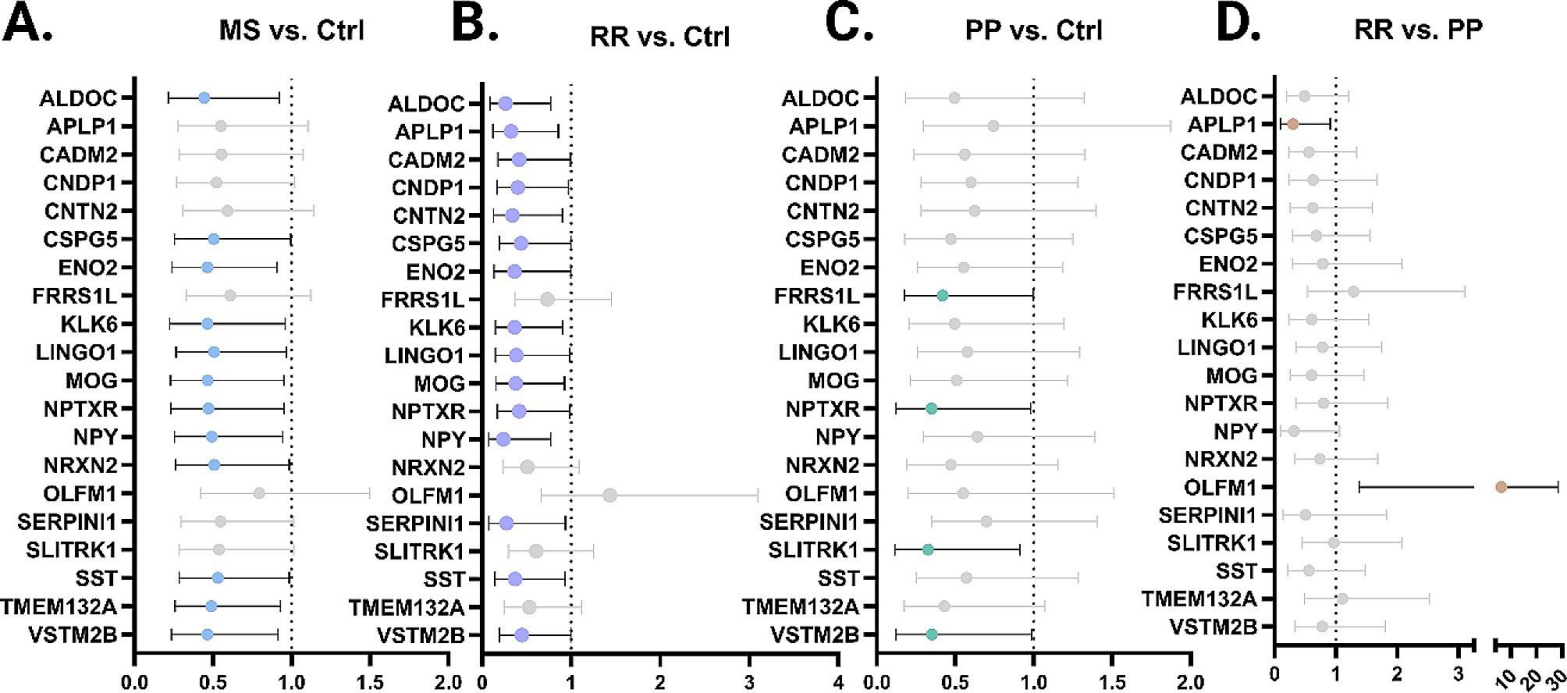
Odds ratio for each listed comparison on scaled, age-adjusted data. Odds ratio displayed for select proteins under each comparison, either MS vs. Ctrl **(A)**, RR vs. Ctrl **(B)**, PP vs. Ctrl **(C)** or RR vs. PP **(D)**. Gray color indicates non-significance. Displayed is the OR with 95% confidence range. Reference line of OR = 1 also displayed.

We next performed a logistic regression for each protein detected in this study to determine if the concentration could distinguish headache control from disease or disease state. Prior to logistic regression, all proteins were scaled as a group and adjusted for age. Indeed, we found 12 significant proteins between MS vs. Ctrl, 15 for RR vs. Ctrl, 4 for PP vs. Ctrl, and 2 for RR vs. PP (Fig. 3; Table 3). Several proteins had area under the curve (AUC) for the receiver operating curve (ROC) of greater than 0.7 for MS vs. Ctrl including ALDOC (AUC = 0.714), LINGO1 (0.721), NPTXR (0.700), SST (0.713), TMEM132A (0.732), and VSTM2B (0.716).

A smaller subset of proteins showed discrimination potential between PP and Ctrl patients after adjusting for age. These include FRRS1L, NPTXR, SLITRK1, and VSTM2B. This analysis also revealed new candidates for biomarkers of disease subtypes with APLP1 and OLFM1 showing strong significant differences between RR and PP. Interestingly, these candidates have opposite expression patterns such that RR patients have a lower mean average of APLP1 and higher mean average of OLFM1 as compared to PP patients.

**Table 3:** Odds ratio for each listed comparison on scaled, age-adjusted data. Logistic regression was performed on all proteins that were scaled and age-adjusted. Listed values indicate significance (*p* < 0.05). Odds Ratio (OR) and area under the curve (AUC) of the corresponding receiver operator curve are also displayed.

Protein	MS vs. Ctrl			RR vs. Ctrl			PP vs. Ctrl			RR vs. PP		
	OR	*p*	AUC	OR	*p*	AUC	OR	*p*	AUC	OR	*p*	AUC
ALDOC	0.444	0.03	0.714	0.264	0.02	0.843	--	--	--	--	--	--
APLP1	--	--	--	0.325	0.02	0.804	--	--	--	0.304	0.03	0.915
CADM2	--	--	--	0.417	0.05	0.775	--	--	--	--	--	--
CNDP1	--	--	--	0.400	0.04	0.793	--	--	--	--	--	--
CNTN2	--	--	--	0.341	0.03	0.811	--	--	--	--	--	--
CSPG5	0.504	0.05	0.691	0.439	0.05	0.786	--	--	--	--	--	--
ENO2	0.464	0.03	0.684	0.366	0.05	0.782	--	--	--	--	--	--
FRRS1L	--	--	--	--	--	--	0.419	0.05	0.850	--	--	--
KLK6	0.464	0.04	0.686	0.367	0.03	0.796	--	--	--	--	--	--
LINGO1	0.506	0.04	0.721	0.385	0.05	0.811	--	--	--	--	--	--
MOG	0.466	0.04	0.689	0.380	0.03	0.800	--	--	--	--	--	--
NPTXR	0.471	0.04	0.700	0.416	0.05	0.789	0.349	0.05	0.832	--	--	--
NPY	0.492	0.03	0.691	0.238	0.02	0.829	--	--	--	--	--	--
NRXN2	0.508	0.05	0.688	--	--	--	--	--	--	--	--	--
OLFM1	--	--	--	--	--	--	--	--	--	6.275	0.02	0.938
SERPINI1	--	--	--	0.275	0.04	0.804	--	--	--	--	--	--
SLITRK1	--	--	--	--	--	--	0.327	0.03	0.850	--	--	--
SST	0.531	0.05	0.713	0.370	0.03	0.800	--	--	--	--	--	--
TMEM132A	0.490	0.03	0.732	--	--	--	--	--	--	--	--	--
VSTM2B	0.465	0.03	0.716	0.445	0.05	0.768	0.351	0.05	0.846	--	--	--

### Neurofilament light is increased in relapsing remitting patients

**Fig. 4:**
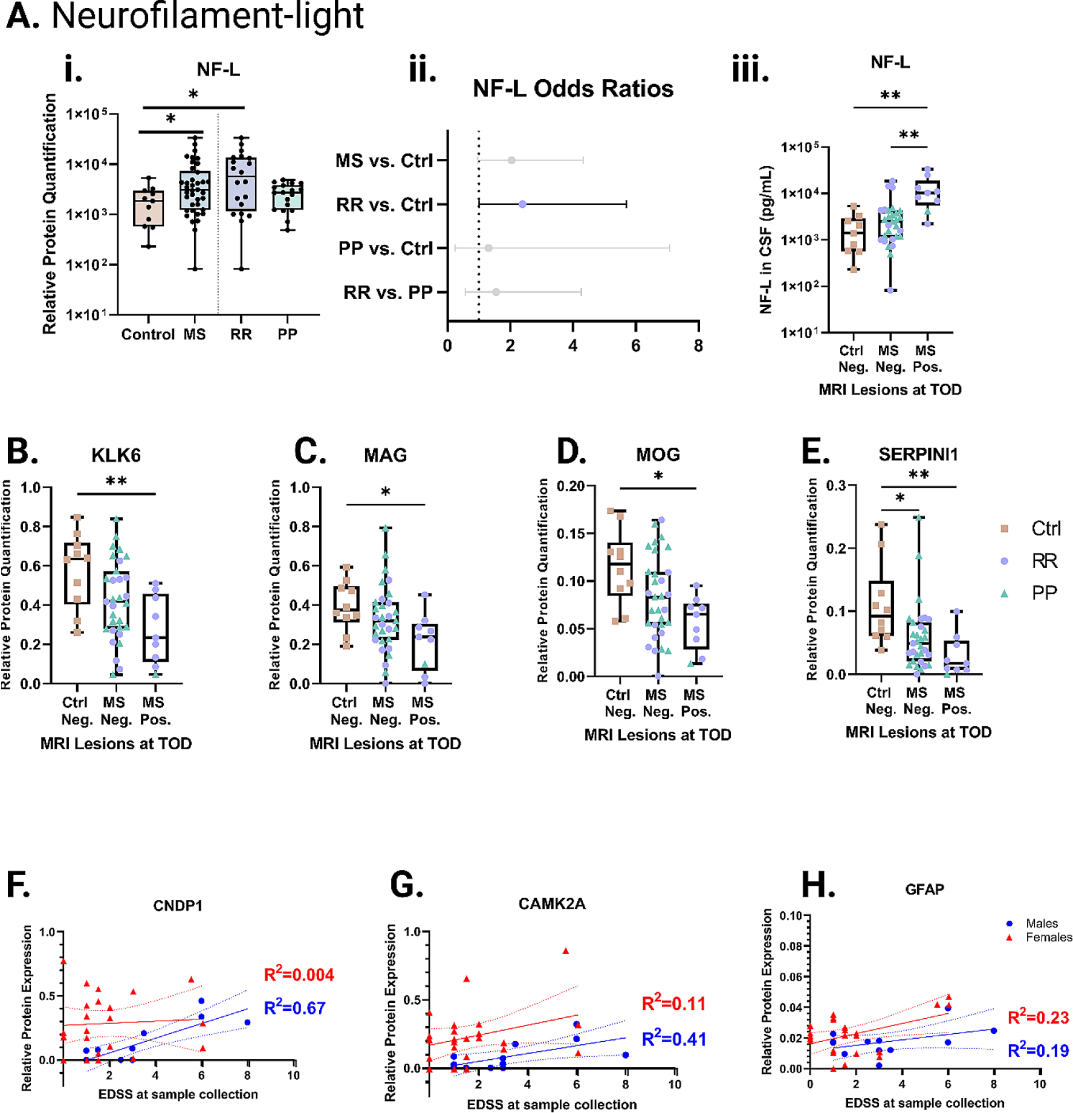
Relation of PRM protein panel to clinically useful biomarkers. Relation of select proteins to clinically useful biomarkers, including NF-L **(A)**, enhancing lesions on MRI **(B-E)**, or EDSS **(F-H)**. **(Ai)** Quantification of NF-L across control, MS, or RR and PP. **(Aii)** Odds ratio for NF-L predicting each comparison via logistic regression. Gray bars indicate non-significant difference. Error bars to 95% CI. **(Aiii)** NF-L quantification between Control-MRI negative, MS-MRI negative, and MS-MRI positive enhancing lesions at time of draw. Other proteins that are differentially expressed are displayed including **(B)** KLK6, **(C)** MAG, **(D)** MOG, and **(E)** SERPINI1. R^2^ (percent of variability explained by the model) calculated via linear regression of **(F)** CNDP1, **(G)** CAMK2A, or **(H)** GFAP are displayed with 95% confidence intervals for either males (blue circles) or females (red triangles). *p < 0.05, **p < 0.01, ***p < 0.001 via Kruskal-Wallis with Dunn multiple comparison corrections.

In addition to the PRM method for quantification of specific peptides, we also measured neurofilament light chain (NF-L) for each of the study participants using an alternative method. A measurable amount was detected in 11/14 controls, 20/20 of the RR, and 18/20 of the PP. The NF-L values spanned three orders of magnitude from a minimum concentration of 82 pg/mL to 33,571 pg/mL. This is consistent with other studies on NF-L [20]. Comparing the NF-L concentrations across disease subtypes, we found that NF-L was significantly higher in patients classified as RR as compared to controls (Fig. 4Ai). There was a trend toward increase in patients with PP, however, this was not significant. Comparison between all MS individuals to Ctrl shows NF-L significantly elevated. When scaled and adjusted for age, NF-L levels could discriminate RR vs. Ctrl patients with an odds ratio of 2.39 (CI = 1.00-5.69, AUC = 0.80, *p* < 0.5, Fig. 4A.ii).

We next examined how NF-L associates with active disease as defined by at least one MRI enhancing lesions at the time of CSF draw (TOD). Included in this analysis are 10 headache control patients with documented negative MRI (Table 1). We found NF-L was significantly higher in MS patients who had a reported MRI enhancing lesion at the time of CSF draw (Fig. 4A.iii). No differences were found in levels of NF-L in patients with positive vs. negative oligoclonal band status (OCB) or kappa light chain status (data not shown).

### Clinical and imaging features correlate with select proteins

In addition to NF-L, we discovered 22 other peptides had concentrations significantly different in a three-way Kruskal-Wallis comparison between Control-MRI negative vs. MS-MRI negative vs. MS-MRI positive at TOD (Table 4). In most cases, these effects were driven by difference between Control-MRI negative vs. MS-MRI positive as determined by Dunn multiple comparison analysis. No protein other than NF-L could distinguish MRI positive vs. negative in the MS population. The list of proteins differentially regulated across disease and MRI status includes proteins highly expressed in myelin-producing oligodendrocytes, such as KLK6, MAG, and MOG (Fig. 4B-D), as well as neuron-enriched proteins like CAMK2A and SERPINI1 (Fig. 4E) [21].

We also utilized EDSS scores gathered at TOD. Interestingly, we did not see a correlation between protein expression with disease severity, including NF-L. However, when we analyzed each sex separately, we noticed GFAP weakly correlates with the female population and ALDOC, CAM2KA, CBLN2, CNDP1, ENO2, LINGO1, and RAB3A positively correlates with the male population (Table 5). CNDP1 boasts the strongest correlation with an R^2^ = 0.67.

**Table 4:** Average protein quantified in patients with and without MRI enhancing lesions at time of CSF draw. Median protein quantified in three groups of patients: Control-MRI negative, MS-MRI negative, and MS-MRI positive enhancing lesions at time of draw. Only proteins with *p*-value < 0.05 by Kruskal-Wallis comparison are listed.

Gene Name	Ctrl MRI Neg.	MS MRI Neg.	MS MRI Pos.	*p*
ALDOC	1.153	0.689	0.397	0.01
APLP1	0.350	0.274	0.146	0.04
CAMK2A	0.295	0.213	0.072	0.03
CAMK2B	0.468	0.299	0.097	0.04
CBLN2	0.025	0.016	0.009	0.02
CSPG5	2.086	1.354	0.621	0.03
ENO2	0.044	0.034	0.019	0.04
KLK6	0.636	0.418	0.233	0.01
LINGO1	0.477	0.246	0.141	< 0.01
LRRC4B	0.174	0.108	0.065	0.04
MAG	0.375	0.319	0.238	0.04
MOG	0.118	0.083	0.065	0.02
NPTX1	0.315	0.200	0.062	0.050
NPTXR	0.234	0.170	0.116	0.02
NPY	0.128	0.056	0.029	< 0.01
NRXN2	2.696	1.404	0.751	0.01
RTN4RL2	0.739	0.461	0.249	0.03
SERPINI1	0.092	0.049	0.018	< 0.01
SLITRK1	0.047	0.028	0.015	0.02
SST	0.075	0.026	0.012	< 0.01
TMEM132A	0.003	0.001	0.001	< 0.01
VSTM2B	0.001	0.001	0.000	0.02

**Table 5:** Sex specific EDSS correlations with proteins of interest. Sex-specific EDSS correlations with proteins of interest that are significant either in the female-only or male-only population subset. R^2^ Goodness of Fit by linear regression. *P* < 0.05 are bolded and indicate slope significantly different than zero.

Gene Name	Females		Males	
	*R* ^2^	*p*	*R* ^2^	*p*
ALDOC	---	---	0.3506	**0.043**
CAM2KA	---	---	0.4138	**0.024**
CBLN2	---	---	0.3586	**0.040**
CNDP1	---	---	0.6723	**0.001**
ENO2	---	---	0.3718	**0.035**
GFAP	0.2296	**0.024**	---	---
LINGO1	---	---	0.3736	**0.035**
RAB3A	---	---	0.4109	**0.025**

### Candidate proteins are highly interconnected

From the set of 65 target peptides, we identified 30 proteins relevant to the disease of multiple sclerosis, whether that be as potential biomarkers for disease, identification of disease subtype, correlation with disease severity, or association with commonly used clinical imaging markers. These 30 proteins are summarized with their observed or predicted subcellular localization [16] (Fig. 1C). This set of proteins spans several regions, ranging from transcription factors predominantly found in the nucleus to transmembrane associated proteins to extracellular secreted proteins. Yet, this group of brain-enriched proteins share the associations with multiple sclerosis described herein. Thus, although this list is relatively small, we figured there would be enriched ontologies in this set, so we performed a String.db search.

**Fig. 5:**
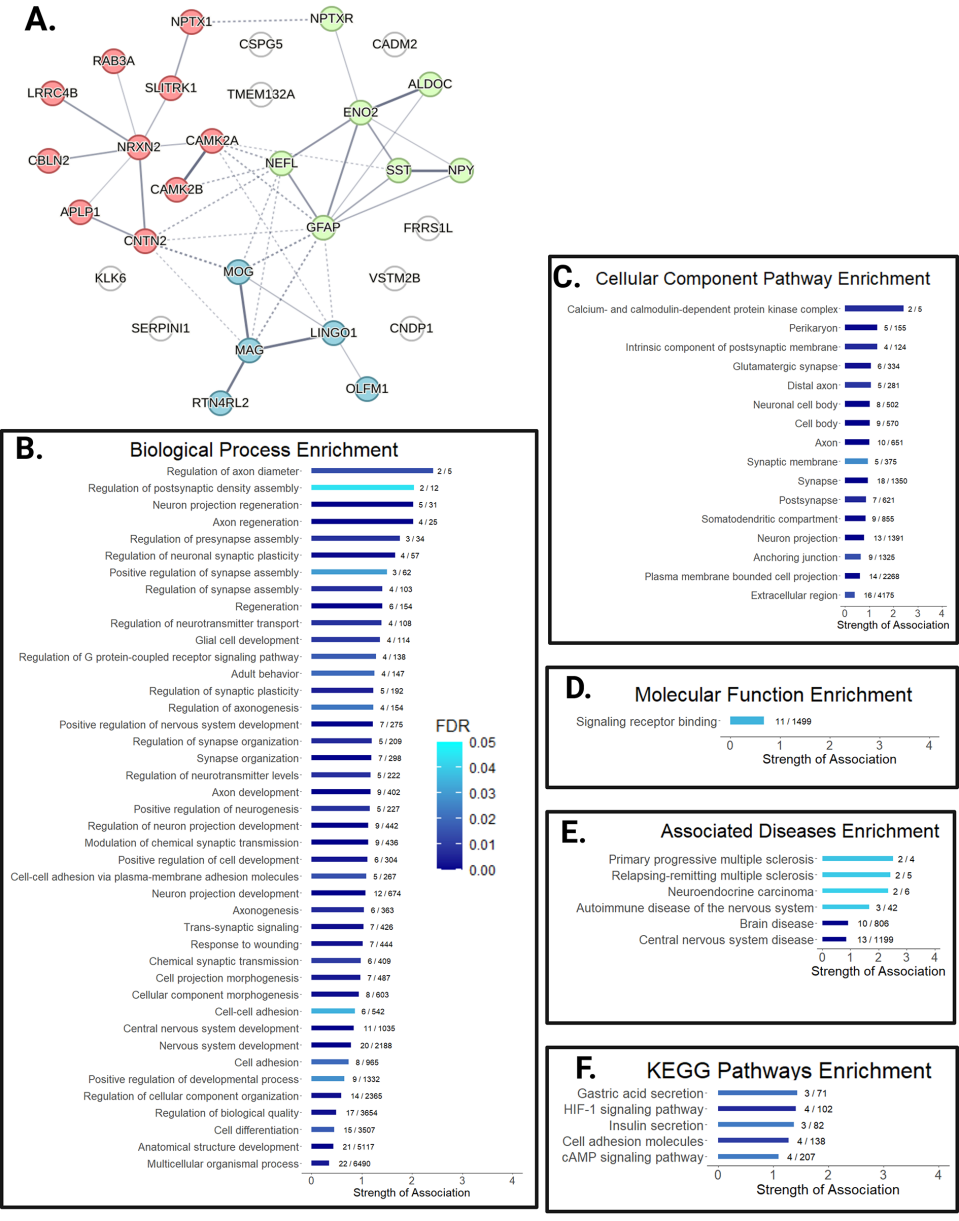
Candidate proteins are highly interconnected and enriched in pathways relevant to MS disease. Analysis of the 28 candidate proteins in the String database. **(A)** Visual mapping of the protein interconnectedness. Color represents cluster by k-means clustering. Confidence of association between proteins is indicated by line thickness. Enrichment plots of **(B)** Biological Processes, **(C)** Cellular Component, **(D)** Molecular Function, **(E)** Associated Disease, and **(F)** KEGG Pathways. Bar indicates strength of association; FDR given by color; fraction indicates observed gene count / background gene count for that pathway.

As suspected, we discovered a tight network with a protein-protein interaction enrichment *p*-value of < 1.0 × 10^6^ (Fig. 5A). The central nodes, including neurofilament light (NEFL), GFAP, MOG, MAG, CAMK2A/B, represent well studied markers of disease. Using k-means clustering, three natural regions emerge. Analyzing all proteins together show the strongest biological processes enriched in this set are related to axon diameter, postsynaptic density assembly, neuron projection, and axon regeneration (Fig. 5B). For cellular component enrichment analysis, CAMK2A and CAMK2B drive the strong hit for calcium-dependent protein kinase complex enrichment in Fig. 5C. Further down the list, it is important to note that over half (*n* = 18) of the proteins are involved with the synapse. All diseases enriched in this set are neurological diseases, with primary progressive and relapsing remitting multiple sclerosis ranking the highest (Fig. 5E). KEGG Pathways enriched in this protein set include “HIF-1 signaling” represented by ALDOC, CAMK2A, CAMK2B, and ENO2 as well as “insulin secretion” represented by RAB3A, CAMK2A, CAMK2B (Fig. 5F).

## Discussion

Using a proteomic analysis approach, we explored a set of 63 CSF protein targets to demonstrate that 30 of these correlated with MS disease state, disability status within sex, and/or MRI imaging features. Of particular significance was the discovery that low levels of APLP1 and CNDP1, or high levels of OLFM1, may distinguish relapsing remitting MS patients from primary progressive MS. This positions these proteins as novel biomarkers for disease management and risk stratification. Further, CNDP1 also correlated with disease severity, especially in males. Finally, we independently validated NF-L as a biomarker for RRMS as well as for MRI enhancing lesions. Other proteins, such as the oligodendrocyte-rich KLK6 protein, also shared this characteristic of deviation from control in active disease.

### Global downregulation of brain enriched proteins in MS CSF

All proteins demonstrating significant changes in the PRM assay from this study had lower abundance in the CSF of individuals with multiple sclerosis as compared to headache controls. This was surprising given that other biomarkers of MS, namely the cytoskeletal proteins NF-L and GFAP, are increased in MS CSF, presumably through disease-related tissue leakage or injury [22]. Examining these discrepancies may reveal insights into our selected cohort and the panel of proteins examined. For example, our finding that GFAP is not increased in MS CSF may reflect the severity of disease at the time of draw, especially since we observed GFAP positively correlates with EDSS in females. Further, our results may reflect the functional importance of the panel of proteins selected for normal CNS maintenance and function [10–13]. Our panel includes proteins crucial for axonal processes, synaptic maintenance, myelin function, and cellular signaling (Fig. 5). In the closely related study, Sohaei et al. (2023) observed a similar damping of the same panel of proteins in a unique MS cohort and cited multiple examples of depleted CSF in CNS diseases [13, 23–30]. For example, Stoop et al. (2017) compared the proteome of clinically isolated syndrome (CIS) with controls and found most differentially abundant proteins had lower expression in CIS. Many of the altered proteins were gray matter related including APLP1 [23]. Similarly, Dhaunchak et al. (2011) observed dysregulation of axoglial molecules in pediatric patients with acquired demyelinating syndrome, an event that often occurs early in MS pathogenesis [27]. These studies, in addition to those showing decreased global expression in frank neurodegenerative conditions like Huntington’s Disease [29], bolster the hypothesis that continued and sustained neurodegeneration from the very start of disease is the basis for MS progression. Thus, our results may represent a snapshot of the neuronal dysfunction.

#### CSF proteins discriminating RR vs. PP

In this study, we observed reductions in CNDP1 in individuals with RR as compared to headache controls or compared to PP. The reductions of CNDP1 protein we observed parallels reports of reduced enzymatic activity in cases of multiple sclerosis, Parkinson’s disease, and cerebrovascular disease [31]. Interestingly, low levels of CNDP1 show some specificity for MS as it can be used to distinguish genuine disease from the mimic clinically isolated syndrome [32, 33]. However, in this prior study, the authors did not distinguish between RR or PP subtypes. Here, we extend the significance of CNDP1 to that manifestation of clinical disease in MS by attributing the low levels of CNDP1 observed in MS patients predominantly to RR subtype in contrast to the near normal levels observed in PP.

CNDP1, carnosine dipeptidase 1, is a metallopeptidase highly expressed in the brain responsible for the cleavage of Xaa-His dipeptides including the preferred substrate carnosine (Xaa = β-alanine) or homocarnosine (Xaa = N-Methyl β-alanine) [34]. Alteration of CNDP1 expression or activity can result in neurological disease, in part due to the dyshomeostasis of the better-studied substrate carnosine. For years, carnosine has been examined for its anti-oxidant properties [35, 36], its dysregulation in psychological/neurological diseases [37, 38], and its promise as a supplemental therapy for neurodegenerative conditions, including Alzheimer’s disease [39, 40] and Parkinson’s disease [41]. These results are recently reviewed by Schon et al. [42]. This protease-substrate relationship complicates the biology as even small changes in CNDP1 have the potential to radically modulate local levels of the neuroprotective carnosine. Further, carnosine cleavage results in free β-alanine, which can act as a partial antagonist of GABA receptors thereby altering brain circuitry [43].

Since CNDP1 is largely expressed by oligodendrocytes in the human CNS, the reduction we observe in RR patients could be due to pathological destruction of the myelin and oligodendrocytes in multiple sclerosis [21]. This raises the potential for CNDP1 to serve as a generic marker of myelin insult, which includes neurodevelopmental diseases, cerebrovascular incident, and leukodystrophies. Mechanistically, the loss of CNDP1 could be compensatory with reductions in CNDP1 activity increasing the pool of its substrate, carnosine. As an antioxidant, higher levels of carnosine may buffer the increased oxidative stress associated with relapses in multiple sclerosis [44, 45]. Interestingly, carnosine is a capable chelator of copper [46], and higher concentrations of copper have been found in patients with multiple sclerosis [47]. Future studies to parse out the role of CNDP1 in other CNS diseases, especially immune mediated disorders affecting myelin such as MOGAD, NMO, and clinically isolated syndrome are warranted.

Amyloid-like protein 1 (APLP1) also showed potential to discriminate RR vs. PP MS in this study. APLP1 is a member of the amyloid precursor protein (APP) family that boasts features of synaptic adhesion molecules [48]. To our knowledge, APLP1 has not clearly been tied to multiple sclerosis, but amyloid beta levels have been studied. In particular, disease severity in MS is associated with lower levels of amyloid beta [49]. Through proteolytic processing of beta- and gamma-site cleavages, APLP1 can generate amyloid-beta-like peptides (APL1beta) that could be used as a surrogate marker of amyloid beta-42 (AB42) [50]. Our representative APLP1 peptide falls immediately after the alpha secretase splice site [51, 52], so it is not clear if the detection of APLP1 would indicate altered AB42 production.

We also examined how OLFM1 can predict disease subtype of multiple sclerosis. Like APLP1, OLFM1 is an understudied player in multiple sclerosis, yet it demonstrates relevant and interesting neurobiology. OLFM1 is a glycoprotein secreted by neurons to prevent axonal growth cone collapse by myelin associated inhibitors such as MAG. OLFM1 accomplishes this stabilization by binding Nogo-66 receptor (NgR1) and forcing NgR1 to dissociate from LINGO1 [53]. Thus, the differences in OLFM1 concentration between RR and PP patients could be indicative of the progression of disease and the characteristic neuronal degeneration of PP. In fact, targeting this pathway has been proposed as a treatment option for PP patients [54]. Our findings highlight the necessity of continued study of supporting proteins in this pathway, not just LINGO1 or NgR1.

### CSF proteins altered in RR vs. headache controls

In this study, the comparison of RR vs. control CSF yielded the greatest number of differentially quantified proteins—20 in total. On the one hand, this list of proteins provides support for new findings such as that CAM2KA may predict progression of multiple sclerosis [13]. On the other hand, we substantiated previously identified proteins relevant to multiple sclerosis, including LINGO1, which is being explored as a potential therapeutic target to improve remyelination [55, 56].

Kallikrein 6 (KLK6) is another example of a protein that is depleted in CSF with disease, not only in RR MS patients compared to control but also in those with MRI enhancing lesions as compared to controls. KLK6 is a secreted serine protease abundantly expressed by oligodendrocytes, long implicated in CNS disease, especially multiple sclerosis [57–61]. The observed downregulation of KLK6 in this study could be related to the acute loss of the mature, myelinating oligodendrocytes. Notably, KLK6 is further lowered in CSF of clinically definite MS patients as compared to clinically isolated syndrome, perhaps due to the proximity to clinical attacks or demyelinating lesions at the time of sample collection [32]. Alternatively, KLK6 might be suppressed in disease to facilitate myelin repair. KLK6 contributes to the protease cascade that participates in MS pathogenesis [62], can degrade myelin proteins [63], and blocks myelination pathways [64]. The general reduction of KLK6 at relapsing stages of disease may foster myelin regeneration.

Relatedly, SERPINI1, a member of the serpin family of serine proteinase inhibitors is lower in MS and RR-MS patients compared to controls (Fig. 1B, C). SERPINI1 is also lower in individuals with MRI lesions than controls. During these inflammatory events that produce rapid bursts of myelin destructive proteases like KLK6, SERPINI1 could become easily overwhelmed. Since SERPINI1 is produced predominantly by neurons, neurodegeneration with late-stage disease may further limit remyelination and repair potential. These data hint at proteases and their inhibitors as positive and negative modulators of multiple sclerosis disease [62].

#### CSF proteins discriminating PP vs. headache controls

Four distinct proteins were able to differentiate PP from headache controls. Notably, three of the four proteins, FRRS1L, NPTXR, and SLITRK1 are important for synaptic function and may serve as markers of synaptic dysfunction [65–67]. FRRS1L (ferric chelate reductase 1-like) forms part of the AMPA receptor complex, and certain genetic variants of *FRRS1L* can result in an epileptic-dyskinetic encephalopathy [68]. The observed decrease in PP MS may contribute to the cognitive and motor dysfunction associated with disease. Similarly, NPTXR (neuronal pentraxin receptor) and its family members, NPTX1 and NPTX2, are abundantly expressed in the synapse and interact with AMPA receptors [69, 70]. Like other pentraxins, including C-reactive protein, neuronal pentraxins can direct the innate immune system to prune synapses [69, 71]. Such a powerful mechanism may easily go awry in the setting of disease resulting in improper synaptic destruction and eventual neuronal degeneration, a hallmark of PP MS.

#### Identified pathways of disease

To summarize the discrete findings in this preliminary study and glean biological insight into the disease process of multiple sclerosis, we utilized the String database [17]. Many of the ontologies involving axons and their synapses were highly enriched in our set of candidate markers of disease. This likely reflects a combination of biological change and our selection of brain-enriched proteins. Interestingly, HIF-1 signaling KEGG pathway was enriched due to the presence of ALDOC, CAMK2A, CAMK2B, and ENO2. HIF-1 signaling is hypothesized to link reactive oxygen species (ROS) and the degenerative processes of autophagy [72]. This is important because the hallmark substrate of multiple sclerosis damage is the lipid-rich, and ROS-sensitive, myelin sheaths of oligodendrocytes. Indeed, oxidative damage is more specific to MS than other inflammatory or neurodegenerative conditions [44]. These damages may trigger HIF-1 signaling, which induces alteration in ALDOC and ENO2 expression, among other glycolytic genes [73]. Changes in glucose metabolism have been noted in the lesions of multiple sclerosis and may alter T-cell response [74, 75]. With this context, our finding that ALDOC and ENO2 are reduced in relapsing remitting disease may reflect alterations in glucose processing and may serve as indicators of dysfunction. Relatedly, in our small study, we also found “insulin secretion” was an enriched KEGG pathway, further tying glucose metabolism and multiple sclerosis. Indeed, there are reports that patients with MS have a high incidence of insulin resistance and severity of resistance correlates with degree of disability and time from diagnosis [76]. While we acknowledge we are underpowered to truly examine these types of connections, our findings hint at the utility of our PRM method to capture not only potential biomarkers but snapshots of nuanced pathogenic mechanisms.

#### Limitations

These results and their interpretations are limited by the exploratory nature of this study. The number of samples is sufficient for direct statistical analysis. However, future studies looking at combinatorial effects of these candidate proteins in a multivariate model should include more patients. We acknowledge that we have not corrected for multiple testing as that would be highly conservative due to the strong correlation between the proteins and the scope of this pilot study. The patients included in this study have a wide range of ages from 11 to 80, which may cloud pathogenic changes in CSF proteins with that of normal aging. This concern is mitigated by the fact that many of the same protein candidates maintain significance after the age-adjusted logistic regression. Our study also focuses on a snapshot in time with diagnosis, EDSS, and MRI lesions derived from the point at which CSF was drawn, which precludes the possibility of disease progression, development, or original misdiagnosis. This is especially relevant since CSF was taken at the time diagnosis was still being established. For example, after sample collection, one patient was later reclassified as having MOG antibody associated disease (MOGAD). Future studies could address this by serially monitoring CSF as well as include a larger cohort of close MS mimics, including MOGAD and neuromyelitis optica (NMO). Relatedly, our cohort has a lower EDSS predominance, especially in the female population, which may explain the greater number of associations found in the more balanced male population. Future studies that follow up on these correlations may want to explicitly sample across a range of EDSS scores. Our findings are also limited by the lack of independent validation by another method, such as ELISA. Finally, we acknowledge that while CSF is the closest surrogate to disease tissue in MS, it is not CNS parenchyma. Therefore, the observed protein changes may mirror any pathological change within the tissue, or it may be a distorted reaction to the disease process.

## Conclusions

Our findings provide myriad opportunities for future study into biomarkers of MS and may hint at proteins that mediate pathogenesis. In sum, these results highlight the utility of parallel reaction monitoring as a method of generating novel, potential biomarkers of disease worthy of robust future study. In this study, our PRM panel provided valuable insight into the pathogenesis of multiple sclerosis, including reductions in brain-enriched proteins in RR-MS, and this technique can be generalized to other CNS diseases with purposeful design of PRM panels.

### Electronic supplementary material

Below is the link to the electronic supplementary material.

Supplementary Material 1

## Data Availability

No datasets were generated or analysed during the current study.

## Abbreviations

MS: Multiple sclerosis
CSF: Cerebrospinal fluid
RR: Relapsing remitting multiple sclerosis
PP: Primary progressive multiple sclerosis
SP: Secondary progressive
CIS: Clinically isolated syndrome
RIS: Radiologically isolated syndrome
EDSS: Expanded Disability Status Scale
PRM: Parallel reaction monitoring
LC: Liquid chromatography
CNS: Central nervous system
LC/MS/MS: Liquid chromatography-tandem mass spectrometry
MOGAD: MOG antibody associated disease
NMO: Neuromyelitis optica
NF-L: Neurofilament light chain
KLK6: Kallikrein 6
CNDP1: Carnosine dipeptidase 1
LINGO1: Leucine rich repeat and immunoglobin-like domain-containing protein 1
MAG: Myelin associated glycoprotein
MOG: Myelin oligodendrocyte glycoprotein
APLP1: Amyloid beta precursor like protein 1
OLFM1: Olfactomedin 1
