# Supplementary material for: Identification of brain-enriched proteins in CSF as biomarkers of relapsing remitting multiple sclerosis
Source: Clin Proteomics. 2024 Jun 16;21:42. doi: 10.1186/s12014-024-09494-5 (PMC11181608; doi:10.1186/s12014-024-09494-5)
Supplement: Supplementary file 1 — Supplementary Material 1 [file 12014_2024_9494_MOESM1_ESM.docx]

**Supplementary File 1**

|  | Control | RRMS | PPMS | All Cases |
| --- | --- | --- | --- | --- |
|  | (N=11) | (N=15) | (N=11) | (N=26) |
| **Age at Collection** |  |  |  |  |
| Median | 45 | 38 | 57 | 47.5 |
| Q1, Q3 | 41.5, 48.5 | 17.5, 45.5 | 54.5, 62.5 | 37.2, 55.8 |
| **EDSS at Sample Collection** |  |  |  |  |
| N | 0 | 14 | 8 | 22 |
| Median | NA | 1 | 2 | 1.5 |
| Q1, Q3 | NA | 0.2, 1.5 | 1.4, 3.6 | 1.0, 2.0 |
| **Time to Sample from Onset of Symptoms (yrs)** |  |  |  |  |
| N | 0 | 15 | 11 | 26 |
| Median | NA | 1.4 | 6.1 | 2.8 |
| Q1, Q3 | NA | 0.8, 2.8 | 3.5, 8.9 | 1.1, 6.5 |
| **MRI Enhancing Lesions at Sample Collection** |  |  |  |  |
| N | 8 | 15 | 11 | 26 |
| No | 8 (100.0%) | 11 (73.3%) | 11 (100.0%) | 22 (84.6%) |
| Yes | 0 (0.0%) | 4 (26.7%) | 0 (0.0%) | 4 (15.4%) |

**Table 1**. **Female demographics and descriptive statistics.**  The CSF of 37 female individuals was collected via lumbar puncture for downstream proteomic analysis. Age, expanded disability scale score (EDSS), time to sample collection after symptom onset, and whether a patient had enhancing lesions on MRI are reported.

|  | Control | RRMS | PPMS | All Cases |
| --- | --- | --- | --- | --- |
|  | (N=3) | (N=5) | (N=9) | (N=14) |
| **Age at Collection** |  |  |  |  |
| Median | 64 | 29 | 55 | 51 |
| Q1, Q3 | 44.5, 64.5 | 17.0, 38.0 | 49.0, 59.0 | 38.2, 59.0 |
| **EDSS at Sample Collection** |  |  |  |  |
| N | 0 | 5 | 7 | 12 |
| Median | NA | 1 | 3.5 | 3 |
| Q1, Q3 | NA | 1.0, 1.5 | 3.0, 6.0 | 1.4, 4.1 |
| **Time to Sample from Onset of Symptoms (yrs)** |  |  |  |  |
| N | 0 | 5 | 9 | 14 |
| Median | NA | 1.9 | 3.1 | 3 |
| Q1, Q3 | NA | 0.5, 5.1 | 2.1, 4.1 | 1.3, 4.2 |
| **MRI Enhancing Lesions at Sample Collection** |  |  |  |  |
| N | 2 | 5 | 9 | 14 |
| No | 2 (100.0%) | 1 (20.0%) | 8 (88.9%) | 9 (64.3%) |
| Yes | 0 (0.0%) | 4 (80.0%) | 1 (11.1%) | 5 (35.7%) |

**Table 2**. **Male demographics and descriptive statistics.**  The CSF of 17 male individuals was collected via lumbar puncture for downstream proteomic analysis. Age, expanded disability scale score (EDSS), time to sample collection after symptom onset, and whether a patient had enhancing lesions on MRI are reported.
